# Irisin-Treg crosstalk: unveiling a mechanism in neural cognitive regulation

**DOI:** 10.1080/07853890.2025.2594281

**Published:** 2025-11-30

**Authors:** Renshu Zhan, Jia Yang, Qinxin Wan, Jin Zhang, Li Liu, Jun Jiang, Liqun Mo, Yiping Bai

**Affiliations:** ^a^ Department of Anesthesiology, The Affiliated Hospital of Southwest Medical University, Luzhou, Sichuan Province, China; ^b^ Anesthesiology and Critical Care Medicine Key Laboratory of Luzhou, Southwest Medical University, Luzhou, Sichuan Province, China; ^c^ Department of Pharmacology and Toxicology, University of Mississippi Medical Center, Jackson, MS, USA; ^d^ Department of General Surgery (Thyroid Surgery), The Affiliated Hospital of Southwest Medical University, Luzhou, Sichuan Province, China

**Keywords:** Irisin, regulatory T cells (treg), neurocognitive function, immune regulation, inflammation, synaptic plasticity

## Abstract

**Background:**

Irisin, an exercise-induced myokine, has emerged as a significant player in neural cognitive functions by interacting with regulatory T cells (Tregs). This review synthesizes the expanding research landscape focused on the Irisin-Treg axis, uncovering its critical role in modulating Treg dynamics and neural immune interactions.

**Methods:**

A comprehensive review of the literature was conducted to explore how Irisin promotes Treg proliferation and differentiation, and fine-tunes immune responses essential for maintaining neural integrity and function. The review also examines Irisin’s neuroprotective properties and its potential as a therapeutic agent.

**Results:**

Irisin enhances Treg-mediated immune regulation, thereby influencing cognitive processes by attenuating inflammation associated with neurodegenerative disorders. It also directly promotes neuronal growth and synaptic plasticity by upregulating neurotrophic factors such as brain-derived neurotrophic factor (BDNF). Collectively, these actions mitigate neuroinflammation and improve neurocognitive function. However, the precise molecular interactions between Irisin and Tregs in brain function remain a subject of debate.

**Conclusions:**

The Irisin-Treg relationship holds promise as an innovative therapeutic target for neurocognitive disorders. Future studies are needed to further elucidate the molecular mechanisms underlying their interaction and to rigorously evaluate the therapeutic efficacy and safety of Irisin-Treg targeted therapies. This review underscores the potential for novel treatment paradigms that could revolutionize the management of neurodegenerative diseases.

Irisin is a myokine secreted from skeletal muscle. It is produced by cleavage of the membrane protein FNDC5 (Fibronectin type III domain-containing protein 5) and was discovered as a new hormone secreted into the body. In white adipocytes, it promotes UCP1 expression and brown adipocyte growth [1,2]. Beyond these metabolic functions, this exercise-induced cytokine has become a research focus in obesity, metabolic syndrome, and diabetes [3]. Newly emerging studies propose that Irisin also plays a pivotal role in the modulation of neurocognitive functions, introducing a fresh vantage point for investigations within the field of neurobiology [4]. Regulatory T cells (Tregs), as a critical cell population within the immune system, are recognized for their contribution to neurocognitive function regulation through the maintenance of immune equilibrium [5]. Recent research has suggested an interplay between Irisin and Treg, which may influence multiple facets of neurocognitive function [6,7]. Despite these advances, the precise mechanisms underlying the neurocognitive regulatory effects of Irisin and Treg interactions remain enigmatic [8]. There is an ongoing discourse in the research community regarding whether Irisin exerts its effects directly on the nervous system or indirectly *via* Treg. These unresolved issues highlight the critical need for further inquiry and define clear pathways for future scientific exploration. This review endeavours to collate and dissect the intricate interplay between Irisin and Tregs in the regulation of neurocognitive functions, delving into the specific mechanisms involved. By doing so, we aim to illuminate the complex molecular and cellular interactions that underpin cognitive processes and how they may be manipulated to ameliorate neurocognitive disorders.

Our aspiration is that this focused examination will not only advance our understanding of neurocognitive function but also foster novel perspectives for therapeutic strategies aimed at treating these disorders. Through a detailed exploration of Irisin and Treg interactions, we anticipate identifying potential therapeutic targets that could lead to innovative treatments for Alzheimer’s disease [9], Parkinson’s disease [10], and other related conditions. Furthermore, this research could pave the way for the development of precision medicine approaches that tailor interventions to the specific molecular profiles of individuals, enhancing efficacy and minimizing side effects. Ultimately, we seek to establish a robust theoretical framework that supports the ongoing quest for breakthroughs in the treatment of neurocognitive disorders.

## 1. Association between Irisin and Treg

### 1.1. Irisin promotes the proliferation and differentiation of Treg, enhancing their regulatory role in the immune system

Irisin, a cytokine induced by exercise, has garnered attention for its pivotal role in enhancing the proliferation and differentiation of regulatory T cells (Tregs), thereby augmenting their regulatory functions within the immune system. Regulatory T cells play a crucial role in maintaining immune homeostasis and tolerance by suppressing excessive immune responses against self-antigens and innocuous environmental antigens. The upregulation of irisin through physical exercise offers a mechanistic insight into how lifestyle factors can influence immune regulation [11,12].

Through promoting the expansion and maturation of Treg cells [11], exercise-induced irisin is believed to exert its effects *via* multiple signalling pathways, potentially involving AMP-activated protein kinase (AMPK) activation and its downstream impacts on cellular metabolism and gene expression. The resultant effect is an enhanced population of Treg cells capable of generating potent anti-inflammatory and immunosuppressive effects. Irisin’s impact extends beyond Tregs to encompass broader immune-modulatory effects. Studies suggest that irisin may contribute to maintaining immune balance by modulating the cytokine milieu within the microenvironment, favouring an anti-inflammatory state. This property not only underscores irisin’s role as a mediator of exercise-induced metabolic adaptations but also highlights its potential in regulating immune responses.

This regulatory effect potentially involves the interaction of Irisin with surface receptors on Treg cells, triggering a cascade of intracellular signal transduction that influences Treg immune function and neurocognitive modulation [4]. Upon binding to Treg receptors, Irisin potentially triggers the activation of Janus kinases (JAK), which subsequently phosphorylate signal transducers and activators of transcription (STAT) proteins. This cascade is critical for initiating signalling pathways that regulate gene expression and cellular responses within Treg cells [5]. These phosphorylated STAT proteins dimerize, translocate to the nucleus, and modulate gene expression pivotal to Treg functionality [13]. Notably, the engagement of Irisin with Treg receptors might stimulate the transcription factor FoxP3, which is integral to Treg development and function. This interaction could enhance FoxP3 gene expression through signal transduction pathways, fortifying the immunosuppressive capabilities of Treg [14]. Irisin is also suggested to impact the expression of genes linked to Treg function, particularly those that encode immunosuppressive cytokines such as IL-10 and TGF-β. This potential influence underscores irisin’s role in modulating immune responses through mechanisms that involve regulatory T cells. Understanding these molecular interactions provides insights into how irisin-mediated pathways might contribute to immune homeostasis, potentially offering new avenues for therapeutic exploration in immune-related disorders [15].

**Figure 1.**
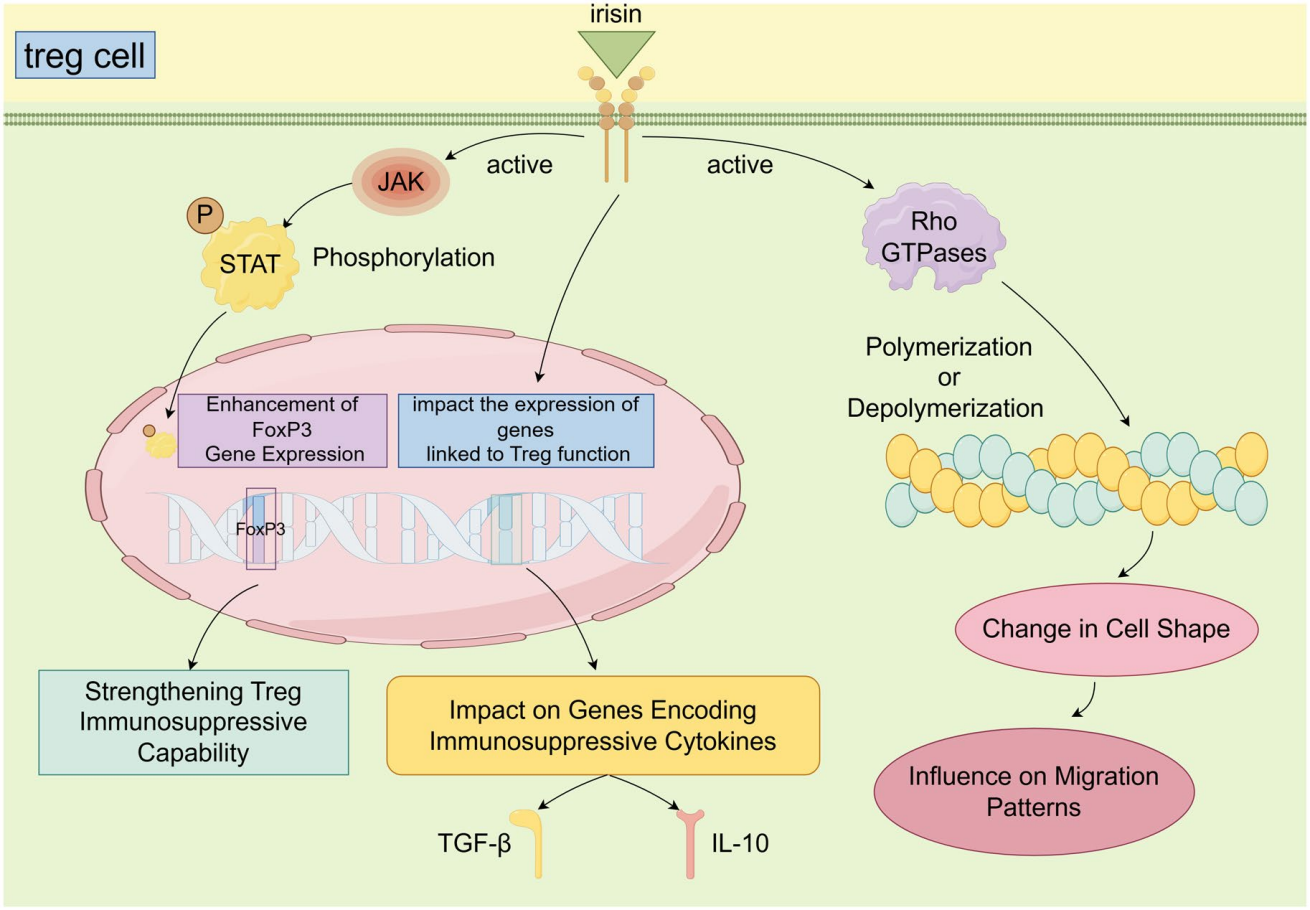
**Irisin-Mediated Regulation of Treg Cell Function and Cytoskeletal Dynamics.** Irisin activates Janus kinases (JAK), which phosphorylate signal transducers and activators of transcription (STAT) proteins. Phosphorylated STAT proteins dimerize and translocate to the nucleus, where they modulate the expression of genes crucial for Treg functionality, including the FoxP3 gene, which is essential for Treg development and function. This process strengthens Treg immunosuppressive capability and impacts the expression of genes encoding immunosuppressive cytokines such as IL-10 and TGF-β. In addition to its effects on gene expression, Irisin influences Treg cell cytoskeletal rearrangements. The activation of Rho GTPases by Irisin leads to the polymerization or depolymerization of actin filaments, resulting in changes in cell shape and migration patterns. These cytoskeletal changes are vital for the positioning and interaction of Treg cells with other immune cells *in vivo*. The figure illustrates the intricate balance between immune regulation and cellular dynamics mediated by Irisin, highlighting its potential as a therapeutic target for immune-related disorders. The interplay between Irisin, Treg cell signaling, and cytoskeletal rearrangements underscores the complex mechanisms by which lifestyle factors, such as exercise, can influence immune homeostasis and response.

Moreover, the cytoskeletal rearrangement within Treg cells, prompted by Irisin stimulation, involves proteins such as actin, which are essential for cell shape, motility, and intracellular transport [16]. The binding of Irisin may initiate the activation of Rho GTPases, leading to the polymerization or depolymerization of actin filaments. This process could subsequently alter the morphology and migration patterns of Tregs. These mechanisms underscore Irisin’s pivotal role in regulating the dynamic behaviour of immune system cells, suggesting its potential to modulate immune responses by affecting Treg cell function. Further exploration of these pathways could unveil novel strategies for manipulating immune responses in the contexts of inflammation and autoimmune diseases [17]. This cytoskeletal reorganization is crucial for the *in vivo* positioning of Treg cells and their engagement with other immune cells [18] (Figure 1).

### 1.2. Irisin creates a more favourable immunological microenvironment for the immunomodulatory action of regulatory T cells (Treg)

Irisin’s capacity to curb the generation of inflammatory cytokines and ameliorate inflammatory responses transcends its immediate physiological impact; more significantly, it fosters a more conducive immunological microenvironment for the immunomodulatory actions of Treg [4,19]. Inflammation is a critical protective response by the body to injury or infection, orchestrated through a complex network of immune pathways to combat potential threats. However, when this response becomes dysregulated or overly activated, it can lead to significant tissue damage and contribute to the development of chronic diseases such as arthritis, cardiovascular disorders, and diabetes. Playing a pivotal role in mitigating these adverse effects are Tregs. Tregs maintain immune homeostasis by modulating the intensity and duration of immune responses. Through cytokine regulation and direct interaction with other immune cells, Tregs precisely control the inflammatory process, thereby preventing pathological damage caused by excessive inflammation. A deeper understanding of the intricate interplay between inflammation and Treg function not only enhances our comprehension of disease pathogenesis but also underscores the potential therapeutic significance of targeting Tregs in the treatment of chronic inflammatory disorders [20]. They achieve this by exerting inhibitory control over the activation, proliferation, and effector functions of various immune cells, such as T cells and B cells, thereby mitigating potential tissue damage and curbing inflammatory responses.

Tregs contribute to inflammation resolution through the secretion of anti-inflammatory cytokines such as interleukin-10 (IL-10) and transforming growth factor-beta (TGF-β), which play crucial roles in attenuating inflammatory processes. Their capability to migrate to inflamed sites and exert localized effects underscores their pivotal role in regulating and terminating inflammatory responses. By maintaining a delicate equilibrium between robust immune defense and excessive inflammation, Tregs ensure that the immune system responds appropriately to external stimuli and internal cues, thereby safeguarding the body against autoimmune diseases and allergies characterized by misguided immune reactions against self-tissues [21].

Tregs play a pivotal role in maintaining immune homeostasis by finely tuning immune responses. Their function extends beyond suppression, instead, Tregs orchestrate intricate interactions and regulatory networks essential for balancing immune activation and tolerance. This role safeguards tissues from excessive inflammatory damage while ensuring effective defense against pathogens and injury. Tregs achieve immune regulation through various mechanisms, including secretion of anti-inflammatory cytokines like IL-10 and TGF-β, direct cell-cell contact mediated suppression, and modulation of dendritic cell function. These multifaceted strategies enable Tregs to restrain effector T cells and prevent autoimmune reactions without compromising immune surveillance against pathogens. Furthermore, recent insights into Treg biology highlight their dynamic nature and plasticity. Tregs can adapt to different tissue microenvironments and respond to specific immunological cues, tailoring their regulatory functions accordingly. This adaptability underscores their versatility in maintaining immune tolerance across diverse physiological contexts. The comprehensive understanding of Treg function underscores their pivotal role in immune regulation and identifies them as promising targets for therapeutic interventions. Strategies aimed at enhancing Treg function or selectively targeting dysfunctional Tregs hold potential for treating inflammatory conditions, autoimmune diseases, and transplant rejection. By elucidating the molecular mechanisms underpinning Treg-mediated immune regulation, ongoing research aims to unlock new avenues for therapeutic development and personalized medicine approaches. In conclusion, the intricate regulatory capabilities of Tregs highlight their significance in immune homeostasis and disease pathogenesis. Continued exploration of Treg biology promises to uncover novel therapeutic strategies harnessing their regulatory potential to improve immune-mediated disorders and enhance overall patient outcomes [22].

Irisin demonstrates a range of mechanisms in modulating inflammatory responses, effectively inhibiting the production of key inflammatory mediators such as tumor necrosis factor (TNF-α), interleukin-1 (IL-1), and interleukin-6 (IL-6) [23]. Molecularly, Irisin engages in direct interactions with the expression of genes implicated in inflammation, exerting regulatory effects through modulation of transcription factor activities and epigenetic mechanisms [24]. This regulatory mechanism dampens the synthesis of inflammatory cytokines, thereby alleviating the intensity of inflammatory responses. Irisin also exhibits the potential to bolster the production of anti-inflammatory factors. This phenomenon is thought to involve the activation of intracellular signalling cascades that facilitate the enhanced expression of genes encoding anti-inflammatory cytokines [25]. These cytokines establish a delicate balance within the immune system, essential for maintaining homeostasis.

Irisin additionally exerts an influence on the modulation of inflammatory signalling pathways, hypothesized to involve interactions with cell membrane receptors. This interaction initiates intricate intracellular signalling cascades that potentially attenuate the efficacy of inflammatory signal transmission. By disrupting the propagation of inflammatory signals through these mechanisms, irisin contributes to the regulation of immune responses and may play a crucial role in mitigating inflammatory-mediated tissue damage. This regulatory function underscores the therapeutic potential of irisin in managing conditions characterized by excessive inflammation, highlighting its significance in biomedical research aimed at understanding and treating inflammatory disorders [26]. It may activate phosphatases to dephosphorylate and reduce the activity of signalling proteins or inhibit kinases, preventing phosphorylation and signal transduction [27]. These enzymatic activities have a profound influence on the activation status of downstream signalling molecules. Irisin might also suppress the expression of inflammatory genes by inhibiting the activation of transcription factors such as nuclear factor-kappa B (NF-κB), thus reducing the intensity of inflammatory responses [28]. This intricate regulatory mechanism empowers Irisin to intricately intervene in inflammatory signal transduction processes, potentially reshaping the spatial and temporal dynamics of signal propagation. Such modulation could critically impact the duration and intensity of inflammatory responses, offering a targeted approach to mitigating inflammatory conditions [29]. This regulatory function is critical for preventing overactivation of inflammatory responses and preserving immune system equilibrium.

Collaboratively, these mechanisms enable Irisin to cultivate an immunological microenvironment that is more favourable for Treg cells [30]. In this enriched context, Treg cells can exert their immunomodulatory effects more efficiently [31]. Tregs are pivotal in restraining exaggerated responses from other immune cells, thereby playing a critical role in maintaining the overall balance of the immune system. This equilibrium is essential for preventing immune-related disorders such as autoimmune diseases and chronic inflammatory conditions. By modulating immune responses, Tregs ensure that the immune system responds appropriately to threats while preventing detrimental overreactions that could lead to tissue damage or autoimmune manifestations.

### 1.3. The potential impact of Irisin and Treg synergy in neural cognitive regulation

In recent years, Irisin has emerged as an exercise-induced cytokine that has attracted considerable interest due to its role in modulating neurocognitive functions [32]. Irisin exhibits a unique capability to permeate the blood-brain barrier, enabling its involvement in a diverse array of central nervous system pathophysiological mechanisms. These encompass neuroinflammation, mitochondrial dysfunction, and protein misfolding, all of which are pivotal in shaping cognitive health outcomes linked to physical exercise. This multifaceted role underscores Irisin’s potential as a mediator in mitigating cognitive decline and neurodegenerative conditions through its intricate interactions within the brain’s physiological milieu [33]. The critical function of Tregs in the immune system is progressively being uncovered [34]. Treg cells play a crucial role in modulating neurocognitive functions by releasing key anti-inflammatory cytokines, notably IL-10 and TGF-β. These cytokines are pivotal in attenuating excessive immune responses and maintaining immune homeostasis. By suppressing inflammation within the central nervous system, Treg cells contribute to a conducive environment for optimal neurocognitive function [35]. The anti-inflammatory nature of Irisin is posited to augment Treg cell functionality, collaboratively fostering the stability of the brain’s internal milieu and preserving normal cognitive and emotional states [36].

**Figure 2.**
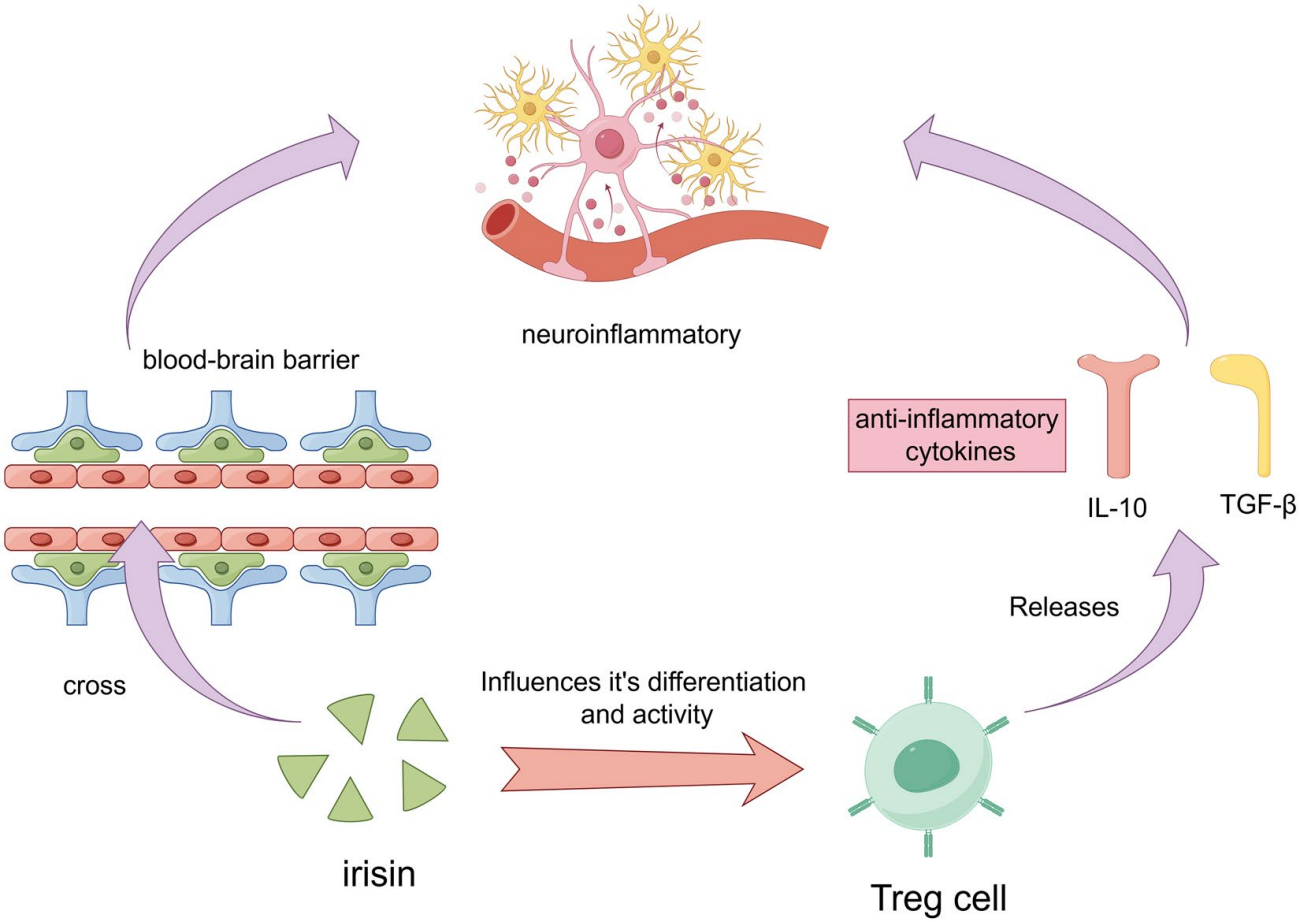
**Irisin and Treg Cells in Neurocognitive Regulation.** Irisin modulates neurocognitive functions by crossing the blood-brain barrier and influencing neuroinflammatory processes. Irisin’s ability to penetrate the blood-brain barrier allows it to interact with central nervous system mechanisms. The function of regulatory T cells (Treg) in the immune system, which release anti-inflammatory cytokines IL-10 and TGF-β. These cytokines help to reduce excessive immune responses and maintain immune homeostasis, contributing to an environment conducive to optimal neurocognitive function. Irisin is proposed to enhance Treg cell functionality, supporting the brain’s internal stability and preserving cognitive and emotional states. The interaction between Irisin and Treg cells suggests a complex biological interplay that affects brain function at multiple levels.

The precise mechanisms underlying the involvement of Irisin and Treg cells in neurocognitive regulation remain incompletely elucidated. Current research suggests that Irisin potentially modulates neuroinflammatory responses by influencing the differentiation and functional activity of Treg cells [7,25] (Figure 2).

The interplay between Irisin and Treg cells potentially implicates intricate signal transduction pathways and intracellular events, whose elucidation is essential for fully understanding their impact on neurocognitive processes [6]. This interaction suggests a sophisticated biological interplay where Irisin and Treg cells operate not only autonomously but also potentially synergistically, exerting influence on brain function across multiple hierarchical levels [37].

Investigating these pathways could reveal how metabolic and immune systems communicate with neuronal circuits, leading to new insights into the cellular and molecular mechanisms that underpin cognitive abilities [38]. For instance, Irisin, recognized predominantly for its involvement in energy metabolism, could potentially engage receptors on neurons or glial cells, initiating signalling cascades that augment synaptic efficacy and plasticity [39]. On the other hand, Treg cells, as modulators of immune response, could play a crucial role in controlling neuroinflammation, a known factor in cognitive dysfunction [40].

Deciphering these intricate pathways necessitates a robust multidisciplinary approach merging neuroscience, immunology, and molecular biology to meticulously chart precise signalling cascades and their ramifications on neural physiology. Leveraging advanced methodologies such as high-resolution imaging, genomics, and proteomics becomes imperative for visualizing and quantifying these complex interactions. These tools are pivotal in unravelling how systemic physiological shifts intricately influence cognitive health and function [41]. This comprehensive understanding could pave the way for novel therapeutic strategies aimed at boosting cognitive function by modulating the activity of Irisin and Treg cells, potentially altering the course of neurodegenerative diseases and cognitive decline [14].

## 2. Impact of irisin on the number and function of regulatory T cells (Treg)

### 2.1. Irisin promotes

**Figure 3.**
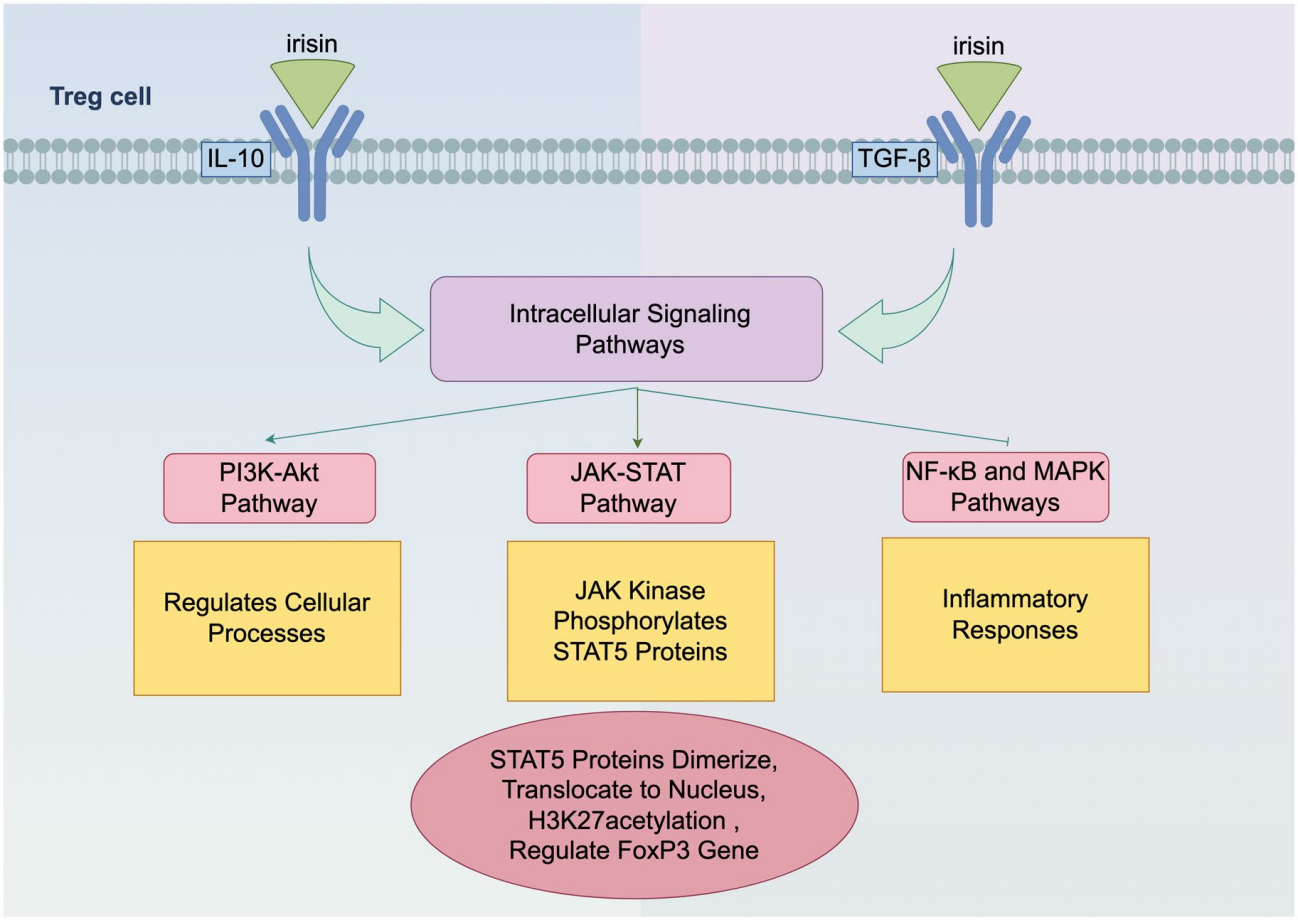
**Irisin-Mediated Activation of Signaling Pathways in Treg Cells.** Irisin binds to surface receptors on Treg cells, including those for IL-10 and TGF-β, thereby initiating intracellular signaling *via* the JAK-STAT, PI3K-Akt, NF-κB and MAPK pathways. Among these pathways, the JAK-STAT pathway is particularly significant. Irisin-activated JAK kinase phosphorylates STAT5, which subsequently dimerizes and translocates to the nucleus. There, phosphorylated STAT5 interacts with acetylated histone H3K27 to cooperatively regulate the expression of the FoxP3 gene, which is essential for Treg cell development and function. Additionally, the PI3K-Akt pathway modulates a variety of cellular processes. Notably, Irisin also inhibits the activity of the NF-κB and MAPK pathways, thereby attenuating the intensity of inflammatory responses. The coordinated actions of these signaling pathways collectively support Treg cell proliferation and function, thereby enhancing the immunosuppressive capacity of Treg cells.

Studies investigating Treg cell proliferation have elucidated Irisin’s capability to bind specifically to surface receptors on Treg cells, including those for IL-10 and TGF-β. This binding initiates robust intracellular signalling cascades, such as the JAK-STAT, PI3K-Akt, NF-κB and MAPK pathways, which are pivotal in regulating cellular responses. Consequently, these pathways contribute significantly to the amplification of Treg cell populations and the augmentation of their immunosuppressive potential [42,43]. The JAK-STAT pathway plays a crucial role in this process, with Irisin-activated JAK kinase phosphorylating STAT5 proteins, which then dimerize, translocate to the nucleus, and regulate genes related to Treg cell proliferation and immunosuppression [44]. Notably, Irisin binding activates STAT5, a key transcription factor that directly regulates the FoxP3 gene, and may further enhance FoxP3 expression by modulating histone modifications, such as altering histone H3K27 acetylation [45]. FoxP3 is essential for Treg cell development and function, enhancing their immunosuppressive properties. Concurrently, the PI3K-Akt pathway modulates a variety of cellular processes, including metabolism, protein synthesis, cell cycle progression, and gene expression. Notably, Irisin also inhibits the activity of the NF-κB and MAPK pathways, thereby attenuating the intensity of inflammatory responses [7,46]. The coordinated actions of these signalling pathways collectively support Treg cell proliferation and function, thereby enhancing the immunosuppressive capacity of Treg cells (Figure 3).

### 2.2. Irisin regulates Treg cell metabolism

Irisin exerts a crucial role in Treg cell metabolism by enhancing glucose uptake and utilization. This enhancement is primarily achieved through the upregulation of glucose transporter proteins and the activation of glycolytic pathways. By facilitating increased ATP production, Irisin supports essential Treg cell functions such as intracellular signalling and intercellular communication. Additionally, Irisin-induced metabolic reprogramming promotes the biosynthesis of immunosuppressive cytokines, including IL-10 and TGF-β, pivotal for maintaining immune balance [47]. Metabolically, Irisin-induced adaptations potentially shift Treg cells towards glycolysis, a metabolic pathway known for its efficiency in generating ATP even under hypoxic or energy-restricted conditions. This metabolic switch not only ensures sufficient energy supply for Treg cells but also helps maintain cellular homeostasis by balancing metabolic demand and supply. By favouring glycolytic metabolism, Irisin enables Treg cells to sustain their immunosuppressive phenotype, crucial for modulating immune responses in varying tissue microenvironments [48]. The profound impact of Irisin on Treg cell biology is underscored by its dual role in enhancing glucose metabolism and modulating cytokine production. Beyond its metabolic implications, Irisin plays a pivotal role in orchestrating Treg cell-mediated immune responses, ensuring effective immune regulation and tolerance. This integrated perspective highlights Irisin as a promising target for therapeutic interventions aimed at fine-tuning immune responses in autoimmune diseases and inflammatory conditions [49].

Irisin also impacts Treg cell lipid metabolism by inhibiting fatty acid synthase activity through pathways like AMPK, reducing fatty acid synthesis and accumulation, and alleviating potential inhibitory effects on Treg cell function [50]. Furthermore, Irisin enhances the activity of CPT1, promoting fatty acid oxidation and energy production, aiding Treg cells in maintaining metabolic balance during inflammation and immune challenges [51].

Irisin’s regulation of mitochondrial metabolism in Treg cells involves the promotion of mitochondrial oxidative phosphorylation, activation of the electron transport chain, and increased ATP production, supporting effective immune regulation [52]. Irisin, a myokine released during physical activity, plays a significant role in modulating mitochondrial dynamics, particularly in the processes of fusion and fission. These activities are crucial for maintaining the integrity and functionality of the mitochondrial network. This maintenance is vital as it helps Treg cells adapt to changing energy demands and enables them to respond swiftly to immune signals. This capability underscores the importance of irisin in not only metabolic regulation but also in immune function, highlighting its potential as a target for therapeutic interventions in diseases where mitochondrial dysfunction and immune responses are implicated [53]. Enhanced mitochondrial function further bolsters Treg cell immune regulatory capacity, effectively inhibiting excessive responses from other immune cells like effector T cells [54].

### 2.3. Irisin’s impact on Treg cell function

Irisin’s regulatory effects on Treg cell function are multifaceted. Irisin’s influence on Treg cell function spans multiple dimensions. Apart from its role in cell differentiation and proliferation, Irisin also impacts the dynamic equilibrium of the cytoskeleton, which plays a vital role in maintaining cell shape, structure, and function [55]. The cytoskeleton, composed of various proteins, provides structural support and facilitates cellular movement. By modulating the expression, assembly, and disassembly of cytoskeletal proteins, Irisin can alter the morphology and migratory capacity of Treg cells. For example, Irisin promotes the polymerization of cytoskeletal proteins, which enhances the stability and resilience of Treg cells. This enhancement may contribute to Treg cells’ ability to navigate effectively within the immunological microenvironment. Irisin’s regulatory role extends to the distribution and arrangement of cytoskeletal proteins within Treg cells. This optimization allows Treg cells to interact more effectively with other immune cells, thereby enhancing their ability to exert immunosuppressive functions. Such interactions are crucial for maintaining immune homeostasis and preventing excessive immune responses. Irisin’s ability to modulate the cytoskeleton underscores its significance in shaping Treg cell behaviour beyond mere differentiation and proliferation. Understanding these mechanisms could pave the way for novel therapeutic strategies targeting immune dysregulation and related disorders [56].

The regulation of the cytoskeleton by Irisin is crucial for the precise positioning of Treg cells within the complex immunological microenvironment. The cytoskeleton, a dynamic network of proteins within cells, provides structural support and is integral to maintaining cell shape, structure, and function. By influencing cytoskeletal dynamics, Irisin affects the morphology and migratory capabilities of Treg cells. This modulation enables Treg cells to effectively navigate through tissues and engage in interactions with other immune cells, which is essential for their regulatory functions in immune responses.

Irisin’s impact on the cytoskeleton extends beyond structural maintenance to functional enhancement. Irisin likely promotes the assembly and stabilization of cytoskeletal elements within Treg cells, making them more resilient and capable of maintaining their regulatory roles amidst varying immune challenges. This enhanced cytoskeletal stability allows Treg cells to respond dynamically to cues from their environment, thereby fine-tuning their immunosuppressive functions according to the needs of the immune system. Irisin-mediated cytoskeletal modifications facilitate the spatial organization of Treg cells within lymphoid tissues and inflamed sites. Proper positioning within these microenvironments is crucial for Treg cells to exert their immunoregulatory effects effectively. By optimizing their spatial distribution, Irisin enhances Treg cells’ ability to interact with antigen-presenting cells and modulate the activation and function of effector T cells, thus contributing to immune homeostasis and tolerance. Irisin’s regulation of the cytoskeleton plays a pivotal role in shaping the behaviour and function of Treg cells within the immune system. Understanding these mechanisms not only sheds light on fundamental aspects of immune regulation but also holds potential for developing therapies targeting immune-related disorders where Treg cell dysfunction is implicated [57,58].

We speculate that Irisin may modulate the expression, assembly, and disassembly of cytoskeletal proteins, thereby affecting the morphology and migratory capacity of Treg cells. Irisin’s ability to modulate Treg cell behaviour through cytoskeletal dynamics underscores its pivotal role in shaping immune responses within tissue environments. Further exploration of these mechanisms holds promise for developing novel therapeutic approaches targeting immune-related disorders.

Furthermore, Irisin may influence the epigenetic state of Treg cells, further regulating the stability and longevity of their immunosuppressive functions [59]. Epigenetic states refer to heritable information within cells that can affect cell function by regulating gene expression. Irisin may modulate the epigenetic state of Treg cells, altering the expression levels of genes related to their immunosuppressive functions, thereby further regulating the stability and longevity of these functions [60].

The discovery of Irisin has provided a new perspective on the proliferation and functional enhancement of Treg [61]. Irisin, a myokine induced by exercise, has been shown to modulate immune responses through intricate mechanisms involving the JAK-STAT signalling pathway, particularly STAT5 activation. This pathway plays a crucial role in the function of Tregs, which are known for their immunosuppressive capabilities. Irisin enhances the proliferative capacity of Tregs by stimulating STAT5 phosphorylation and subsequent activation. This activation promotes the expression of FoxP3, a master transcription factor critical for Treg cell development and function. By enhancing FoxP3 expression, Irisin reinforces the immunosuppressive properties of Tregs, thereby dampening excessive immune responses and promoting immune tolerance. This regulatory role of Irisin in enhancing Treg cell function highlights its potential therapeutic implications in autoimmune diseases and conditions characterized by dysregulated immune responses. Further research into the precise molecular mechanisms by which Irisin influences the JAK-STAT pathway and Treg cell biology will be crucial for fully understanding its therapeutic potential [62]. Furthermore, in a mouse model of inflammatory pain, Irisin modulates the cytokine environment, such as increasing the production of IL-10, further enhancing the anti-inflammatory effect of Treg cells, which is crucial for controlling excessive immune responses and maintaining immune homeostasis [63].

The impact of Irisin on Treg cell differentiation involves epigenetic regulatory mechanisms. The influence of Irisin on Treg cell differentiation may be achieved through the precise regulation of epigenetic marks. Specifically, Irisin may affect DNA methylation patterns, particularly the methylation status of promoter regions in Treg cell-specific genes, thereby regulating the expression of these genes [64]. Moreover, Irisin may promote the expression of the FoxP3 gene by affecting histone modifications, such as modulating histone H3K27 acetylation, which is essential for the stability and function of Treg cells [49]. These epigenetic changes provide an additional level of regulation for Treg cells, enabling them to adapt to different immunological environments.

## 3. The role of Irisin and Treg in the regulation of neurocognitive function

### 3.1. Irisin and Treg may influence neurocognitive function by modulating neuroinflammation

Irisin plays a multifaceted role in regulating neuroinflammation and promoting neuroprotection. Primarily induced by exercise, Irisin exerts direct effects by inhibiting the release of inflammatory factors such as TNF-α, IL-1β, and IL-6 within the brain. This inhibition helps reduce the incidence of neuroinflammation, a common feature in many neurological disorders. By mitigating inflammatory responses, Irisin contributes to protecting neurons from inflammatory damage, thereby preserving neuronal function and promoting overall neuroprotection. Further research into Irisin’s precise mechanisms of action and its potential therapeutic applications could provide new insights into combating neuroinflammatory conditions and enhancing brain health [65]. Secondly, the synergistic action of Irisin and Treg cells enhances immunosuppressive capabilities, with Treg cells secreting anti-inflammatory cytokines such as IL-10 and TGF-β, further suppressing excessive immune responses and reducing neuroinflammation [66]. Additionally, Irisin enhances cellular antioxidant capacity by increasing the expression of antioxidants such as SOD, GPx, and GR, mitigating the damage of oxidative stress to neurons [8].

**Figure 4.**
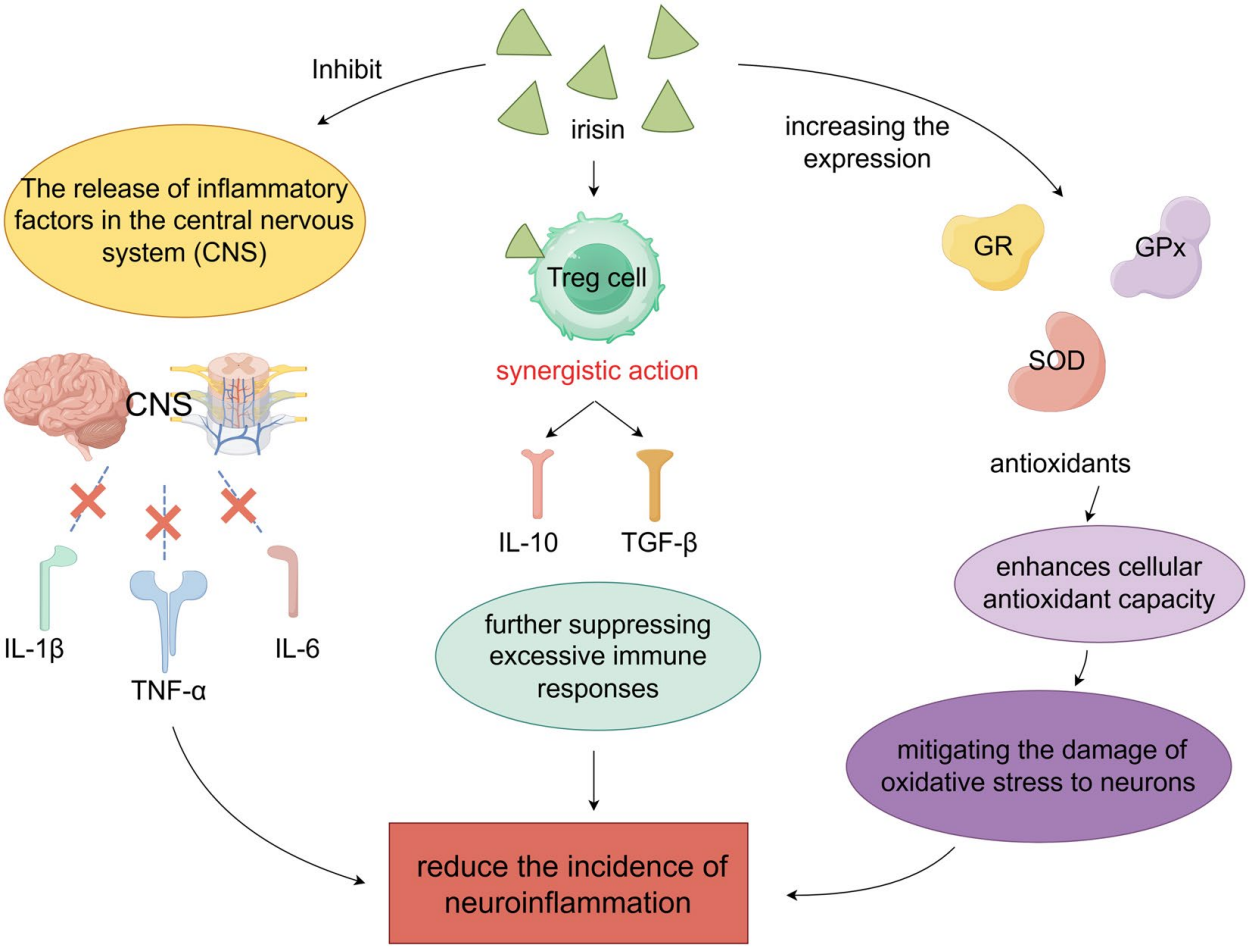
**Irisin and Treg Cells in Neuroinflammation and Neuroprotection: Reducing Inflammation and Enhancing Antioxidant Defense.** Irisin inhibits the release of inflammatory factors such as TNF-α, IL-1β and IL-6 in the central nervous system (CNS), thereby reducing neuroinflammation and protecting neurons from damage. The synergistic action of Irisin and Treg cells enhances immunosuppressive capabilities. Treg cells secrete anti-inflammatory cytokines IL-10 and TGF-β, further suppressing excessive immune responses and contributing to a neuroprotective environment. Additionally, Irisin increases the expression of antioxidants like SOD, GPx and GR, enhancing cellular antioxidant capacity and mitigating oxidative stress to neurons. This dual action of direct inhibition of cytokine release and modulation of intracellular signaling pathways positions Irisin as a promising candidate for therapeutic interventions in neurodegenerative diseases.

Irisin, induced by physical exercise, is increasingly recognized for its role in modulating neuroinflammation through various mechanisms. Beyond directly inhibiting the release of inflammatory cytokines like TNF-α, IL-1β and IL-6, Irisin also affects neuroinflammation-related signalling pathways. Studies have shown that it can attenuate the activation of nuclear NF-κB and MAPK pathways within neurons and glial cells. These pathways are pivotal in the initiation and propagation of neuroinflammatory responses. By reducing the activation of NF-κB and MAPK signalling, Irisin limits the production of inflammatory mediators and decreases the intensity and duration of neuroinflammatory responses [67,68]. This dual action-direct inhibition of cytokine release and modulation of intracellular signalling pathways-positions Irisin as a promising candidate for therapeutic interventions aimed at mitigating neuroinflammation-associated neurodegenerative diseases and promoting neuroprotection [49,69]. Further research into the detailed mechanisms underlying Irisin’s effects on these pathways is essential to fully harness its therapeutic potential in clinical settings. In terms of neuroprotection and repair, Irisin is involved in the repair process following neural injury by promoting the proliferation and differentiation of neural progenitor cells, contributing to the improvement of cognitive function and the recovery from neurodegenerative diseases [70] (Figure 4).

Given the role of Irisin and Treg cells in regulating neuroinflammation, they may serve as potential targets for the treatment of neurodegenerative diseases such as Alzheimer’s and Parkinson’s [71]. Modulating Irisin levels and enhancing the function of Tregs hold promising therapeutic potential in mitigating the progression of neurodegenerative diseases. Irisin, primarily released during physical exercise, has been implicated in enhancing synaptic plasticity and neuroprotection [72]. Future research should delve deeper into the specific mechanisms through which Irisin influences neuroinflammation. This includes its role in modulating inflammatory pathways and cellular responses within the brain. Similarly, Tregs play a crucial role in dampening neuroinflammation through their regulatory effects on immune responses within the brain. They modulate microglial activation, cytokine production, and immune cell infiltration, thereby creating a neuroprotective environment that supports neuronal health and synaptic integrity. Understanding the precise molecular pathways and cellular interactions involved in Treg-mediated neuroprotection is essential for optimizing therapeutic strategies. Clinical studies are imperative to validate the roles of Irisin and Tregs in human neurodegenerative diseases. These studies can elucidate whether manipulating Irisin levels or enhancing Treg function effectively translates into clinical benefits, such as slowing disease progression or preserving cognitive function in affected individuals. Moreover, longitudinal studies are needed to assess the long-term efficacy and safety of interventions targeting Irisin and Tregs in diverse patient populations. Advancing our understanding of Irisin and Treg biology in neurodegenerative contexts may uncover new therapeutic targets. This could pave the way for the development of innovative treatments that not only alleviate neuroinflammation but also promote neuroregeneration and functional recovery in affected brain regions. While the potential of Irisin and Tregs in neurodegenerative diseases is promising, further comprehensive research is essential. Rigorous exploration of their mechanisms, coupled with robust clinical validation, is critical for realizing their therapeutic potential and improving outcomes for patients with these debilitating conditions [73].

Advancing our understanding of Irisin and Tregs in the context of neurodegenerative diseases holds significant promise for developing innovative therapeutic strategies. Irisin, a myokine released during physical exercise, has emerged as a potential mediator of neuroprotection by enhancing synaptic plasticity and reducing neuroinflammation. Its mechanisms likely involve modulation of inflammatory pathways and promotion of neurotrophic factors, which are crucial for neuronal health and function. This suggests that strategies to elevate Irisin levels or mimic its effects could potentially slow disease progression and improve cognitive outcomes in neurodegenerative conditions. Tregs, known for their immunomodulatory functions, play a pivotal role in maintaining immune homeostasis within the central nervous system (CNS). They suppress excessive inflammation by modulating microglial activation, cytokine production, and immune cell infiltration into the CNS. Enhancing Treg function or increasing their numbers has shown promise in experimental models of neurodegenerative diseases, indicating their potential as therapeutic targets to mitigate neuroinflammation and preserve neuronal integrity. Understanding the intricate mechanisms by which Irisin and Tregs exert their neuroprotective effects is crucial. This includes exploring their interactions with specific cellular and molecular pathways involved in neuroinflammatory processes. Insights gained from such research could lead to the development of targeted therapies that specifically modulate Irisin signalling or enhance Treg function to combat neurodegeneration effectively. Clinical studies are essential to validate these preclinical findings and translate them into effective treatments for human patients. These studies will assess the safety, efficacy, and long-term benefits of interventions targeting Irisin and Tregs in diverse neurodegenerative diseases. Longitudinal studies will be particularly valuable in determining the optimal strategies for patient-specific treatment approaches, potentially leading to personalized therapies tailored to the unique biological profiles of individuals with neurodegenerative disorders.

Investigating Irisin and Tregs in the regulation of neuroinflammation offers a promising avenue for developing transformative therapies in neurodegenerative diseases. By deepening our understanding of their mechanisms and conducting rigorous clinical studies, researchers aim to usher in a new era of treatments that could significantly improve outcomes and quality of life for patients affected by these challenging conditions [73].

### 3.2. The potential role of Irisin and Treg in synaptic plasticity regulation

Research has uncovered that Irisin and Treg cells may play pivotal roles in the regulation of neurocognitive functions by influencing synaptic plasticity. Synaptic plasticity refers to the adjustability of synaptic connection strength and efficiency between neurons within the nervous system, serving as the foundational mechanism for cognitive capacities such as learning and memory [74]. These findings suggest that Irisin and Treg cells could modulate the dynamic changes in synaptic strength, which are crucial for the encoding of new information and the adaptation of neuronal networks in response to learning.

Further, it has been proposed that Irisin might enhance synaptic plasticity by promoting the expression of neurotrophic factors, which support neuron survival and growth [75]. Meanwhile, Tregs are implicated in maintaining homeostasis within the neuroinflammatory milieu [39], suggesting a critical role in mitigating inflammation-induced synaptic dysfunction and thereby safeguarding cognitive function. These interactions underscore a complex regulatory network wherein metabolic and immune signals converge, exerting profound influences on brain health and neurocognitive outcomes. The intricate mechanisms through which Tregs modulate neuroinflammation involve the suppression of pro-inflammatory responses, thereby potentially attenuating neuronal damage and cognitive decline. This regulatory interplay highlights the dynamic interrelationship between peripheral immune responses and central nervous system function, offering insights into therapeutic avenues for neuroinflammatory disorders [76].

By expanding our understanding of these mechanisms, researchers hope to unlock new therapeutic avenues that could mitigate or even reverse the impairments seen in neurodegenerative diseases and cognitive decline associated with aging. This insight into the molecular pathways governing synaptic plasticity opens up exciting possibilities for interventions that enhance cognitive resilience and neuroplasticity, ultimately improving life quality for individuals suffering from neurocognitive disorders.

In the realm of synaptic plasticity regulation, emerging research has pointed to Irisin’s potential role in fostering synaptogenesis and enhancing synaptic strength. Experimental evidence suggests that Irisin, a myokine induced by exercise, can influence neuronal growth and development. By promoting the proliferation of dendritic spines and enhancing synaptic connectivity, Irisin may facilitate the formation of robust neural networks critical for cognitive functions such as learning and memory [77]. In an Alzheimer’s disease mouse model, the mechanisms underlying Irisin’s effects on synaptic plasticity involve its interaction with neurotrophic factors and signalling pathways within neurons. Irisin has been shown to upregulate brain-derived neurotrophic factor (BDNF), a key player in synaptic plasticity, which promotes neuronal survival, growth, and synaptic formation. Additionally, Irisin may modulate neurotransmitter systems involved in synaptic transmission, thereby impacting synaptic strength and plasticity [78]. Preclinical studies utilizing animal models have demonstrated that Irisin administration enhances cognitive performance in tasks involving learning and memory [79]. These findings suggest that Irisin’s ability to promote synaptogenesis and improve synaptic function could translate into therapeutic strategies for neurodegenerative disorders and cognitive impairments. Irisin emerges as a promising candidate in the regulation of synaptic plasticity, offering insights into its potential therapeutic applications for enhancing cognitive functions and combating neurological diseases characterized by synaptic dysfunction.

The role of Treg cells in regulating synaptic plasticity primarily hinges on their potent immunomodulatory functions. Tregs exert their influence by suppressing the activation of immune cells and dampening inflammatory responses within the nervous system. This pivotal function serves to attenuate the levels of inflammation that can otherwise induce neuronal damage and exacerbate pathological stimuli. By mitigating inflammatory cascades, Tregs contribute significantly to maintaining the delicate balance of immune responses in neural environments, thereby safeguarding neuronal integrity against various neurotoxic insults and promoting conditions conducive to synaptic plasticity [80].

Inflammatory responses are intricately linked to synaptic plasticity impairment observed in neurodegenerative diseases, underscoring the potential of Tregs to play a pivotal role in restoring and regulating neurocognitive function. Tregs, a specialized subset of CD4+ T cells known for their immunoregulatory functions, exert profound anti-inflammatory effects within the central nervous system (CNS). They achieve this by modulating microglial activation, cytokine production, and immune cell infiltration, thereby attenuating neuroinflammation-a hallmark feature of various neurodegenerative conditions. By mitigating excessive inflammation, Tregs foster an environment conducive to neuronal survival and synaptic remodelling. Tregs are implicated in promoting neuroprotective pathways and enhancing neurotrophic support mechanisms. They interact closely with astrocytes and neurons, influencing the secretion of pivotal neurotrophic factors such as BDNF and glial cell-derived neurotrophic factor (GDNF), both critical for synaptic plasticity and neuronal resilience. Through these interactions, Tregs bolster synaptic connectivity and fortify neurons against inflammatory insults. Experimental evidence underscores Tregs’ potential to ameliorate cognitive decline in neurodegenerative diseases by preserving synaptic integrity. Studies using animal models of Alzheimer’s disease, Parkinson’s disease, and multiple sclerosis have demonstrated that enhancing Treg activity can mitigate synaptic dysfunction and improve cognitive outcomes. These findings highlight Tregs as promising therapeutic targets for restoring synaptic plasticity and halting cognitive decline in neurodegenerative disorders. The immunomodulatory functions of Tregs offer a compelling avenue for addressing neuroinflammation-associated impairments in synaptic plasticity [19,81]. By supporting neurocognitive recovery through their regulatory roles and neuroprotective effects, Tregs present novel opportunities for therapeutic intervention in the management of neurodegenerative diseases. Continued research into Tregs’ mechanisms and their interactions within the CNS promises to advance our understanding and treatment of these challenging conditions.

**Figure 5.**
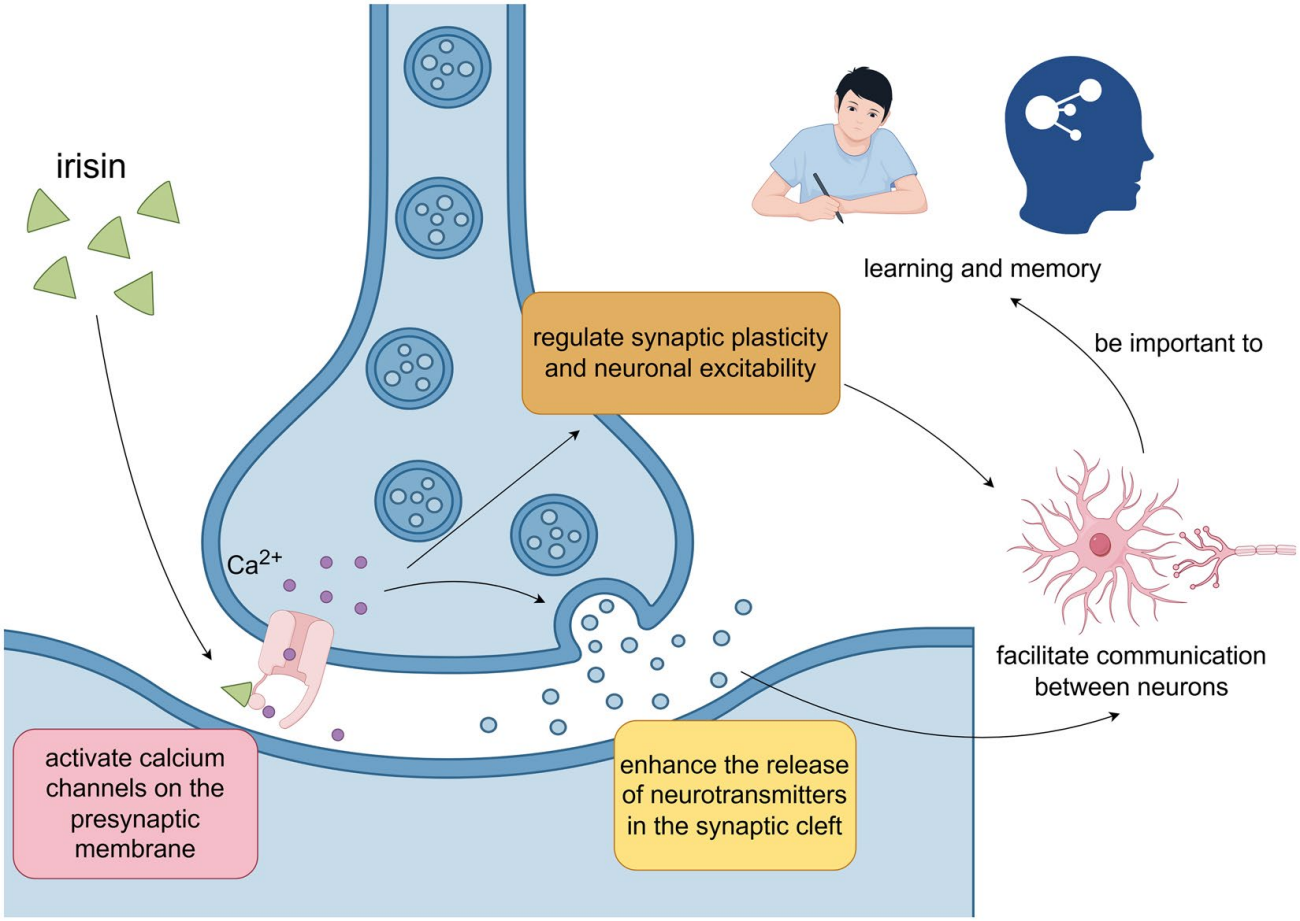
**Irisin’s Role in Synaptic Plasticity and Neuronal Communication.** Irisin contributes to synaptic plasticity and neuronal communication. Irisin activates calcium channels on the presynaptic membrane, which enhances the release of neurotransmitters in the synaptic cleft. This increased neurotransmitter release facilitates communication between neurons and is crucial for learning and memory processes. Additionally, Irisin regulates synaptic plasticity and neuronal excitability, which are essential for the formation and reinforcement of synaptic connections. These connections are the basis for cognitive functions such as learning and memory. The figure also suggests that Irisin’s actions are important for maintaining the dynamic changes in synaptic strength that enable the encoding of new information and the adaptation of neuronal networks in response to learning experiences.

Irisin’s interaction with Tregs represents a critical pathway in the modulation of neurocognitive function, particularly through its influence on synaptic plasticity. Recent studies have demonstrated that Irisin plays a pivotal role in regulating synaptic plasticity by impacting the quantity and functional efficacy of Tregs. Specifically, Irisin exerts a dual effect: firstly, it enhances the population of Tregs, thereby bolstering their ability to modulate immune responses, mitigate inflammatory damage, and alleviate oxidative stress, all of which are detrimental to synaptic plasticity [7,26]. Secondly, Irisin is implicated in direct neurotrophic effects, potentially facilitating neuronal growth and development processes crucial for synapse formation and the reinforcement of synaptic connections [82]. These findings underscore Irisin’s multifaceted role in neurocognitive regulation, highlighting its therapeutic potential in addressing conditions where synaptic plasticity deficits contribute to cognitive impairment (Figure 5).

However, the precise mechanisms through which Irisin and Tregs modulate synaptic plasticity remain incompletely understood and necessitate further investigation. Future research efforts could employ cell culture and animal model experiments to explore how Irisin and Tregs influence synaptic morphology and function. This research could delve deeper into uncovering the underlying molecular mechanisms and signalling pathways involved. Irisin, a myokine induced by exercise, and Tregs, known for their immunomodulatory roles, have both shown potential in preclinical studies to enhance synaptic connectivity and neuronal resilience in conditions marked by neuroinflammation. Understanding their specific roles in synaptic maintenance and plasticity could shed light on novel therapeutic approaches for neurodegenerative diseases and cognitive impairments. Investigating Irisin’s interactions with neurotrophic factors like BDNF and Tregs’ regulatory effects on immune responses within the CNS are critical avenues for research. These studies could provide insights into how these molecules mitigate inflammatory responses and promote neuroprotective environments conducive to synaptic integrity. By elucidating these mechanisms, researchers aim to advance our understanding of neurocognitive function regulation and potentially uncover new strategies for therapeutic interventions targeting synaptic dysfunction in neurological disorders.

### 3.3. Irisin and Treg influence neurotransmitter release affecting cognitive function

The interaction between Irisin and Tregs plays a critical role in modulating neurocognitive functions, particularly through their influence on neurotransmitter dynamics. This modulation is essential for maintaining and enhancing cognitive functions such as learning, memory, and information processing. Irisin interacts with Tregs to regulate immune responses in the brain, which in turn impact neurotransmitter activity. Tregs, known for their immunomodulatory roles, help maintain a balanced inflammatory environment within the brain. This balance is critical for preserving the integrity of neural circuits involved in cognitive processes. The interplay between Irisin and Tregs suggests a complex regulatory mechanism that influences cognitive function. Irisin’s ability to modulate neurotransmitter levels may contribute to improved synaptic plasticity and neuronal health, enhancing overall cognitive performance. Moreover, Tregs’ role in immune regulation within the brain can mitigate neuroinflammation, a factor implicated in various neurodegenerative diseases and cognitive impairments. FNDC5/irisin levels are reduced in the hippocampi of patients with Alzheimer’s disease (AD) and in experimental models, with evidence suggesting that increasing these levels can restore synaptic plasticity and improve memory [78]. Additionally, another study found that Irisin significantly reduces amyloid-β pathology in AD models by enhancing the release of the Aβ-degrading enzyme neprilysin from astrocytes. These findings emphasize the critical role of Irisin in the pathology of AD and indicate its potential as a therapeutic target for both treatment and prevention [83]. Understanding the detailed mechanisms through which Irisin and Tregs interact to regulate neurotransmitter dynamics and immune responses in the brain is crucial for developing targeted therapies. Such therapies could potentially alleviate cognitive decline in conditions like Alzheimer’s disease and Parkinson’s disease, where synaptic dysfunction and neuroinflammation are prominent features.

In conclusion, the synergy between Irisin and Tregs underscores their potential as therapeutic targets for preserving and enhancing neurocognitive functions. Further research into these pathways holds promise for developing innovative treatments that address the underlying mechanisms of cognitive decline in neurological disorders.

In terms of neurotransmitter synthesis, the interaction between Irisin and Treg can promote the activity of enzymes related to neurotransmitter synthesis, thus increasing the amount of neurotransmitters produced [83]. Irisin, a myokine released during exercise, enhances neurotransmitter synthesis and synaptic function in the brain, crucial for learning and memory. It activates specific signalling pathways that increase the expression and activity of enzymes involved in neurotransmitter production. This elevation in neurotransmitter levels within the synaptic cleft improves neuronal communication, fostering synaptic plasticity-the ability of synapses to strengthen in response to activity. By promoting these processes, Irisin potentially enhances cognitive function, offering promising avenues for therapeutic interventions aimed at improving brain health and treating cognitive disorders [84].

The interaction between Irisin, a myokine released during physical exercise, and Tregs plays a pivotal role in regulating neurotransmitter dynamics and consequently influencing cognitive function. Irisin has been shown to enhance the release of neurotransmitters in the synaptic cleft, thereby facilitating communication between neurons essential for cognitive processes such as learning and memory [85]. This effect is mediated partly through Irisin’s ability to activate calcium channels on presynaptic membranes, promoting neurotransmitter release and modulating synaptic plasticity and neuronal excitability. Irisin interacts with Tregs, which regulate immune responses in the brain, influencing neuroinflammatory processes [,^86^]. Tregs help maintain a balanced inflammatory environment that is crucial for preserving neuronal health and synaptic function. Dysregulation of this balance can lead to synaptic dysfunction and cognitive impairments observed in neurodegenerative diseases. The combined actions of Irisin and Tregs highlight their potential as therapeutic targets for enhancing cognitive function and mitigating cognitive decline [87]. By understanding the intricate mechanisms through which Irisin influences neurotransmitter release and how Tregs regulate neuroinflammation, researchers can develop targeted interventions aimed at preserving synaptic integrity and improving cognitive outcomes in neurological disorders. The interplay between Irisin and Tregs represents a promising area of research for advancing our understanding of neurocognitive function regulation and developing innovative therapies for conditions characterized by cognitive decline. Continued exploration of these pathways may lead to transformative treatments that benefit individuals affected by neurodegenerative diseases and other cognitive impairments.

## 4. Controversies in the mechanism of action of irisin, treg, and neurocognitive function regulation

### 4.1. Controversies in the mechanism of interaction between Irisin and Treg cells

The interaction between Irisin and Treg cells and its impact on cognitive function is a highly debated research area, involving multiple biological processes. On one hand, Irisin may indirectly affect cognition by enhancing the immunomodulatory function of Treg cells, which secrete anti-inflammatory cytokines such as IL-10 and TGF-β, helping to reduce neuroinflammation and potentially having a positive impact on cognitive function, highlighting the close interaction between the immune and nervous systems. On the other hand, Irisin has also shown direct effects on neurons [88], potentially participating in the modulation of neuroprotection and synaptic plasticity by promoting the expression of neurotrophic factors such as BDNF, affecting cognitive processes [33]. Synaptic plasticity, as the basis for learning and memory formation, the direct impact of Irisin on it may help improve cognitive function in neurodegenerative diseases.

Furthermore, the regulatory effect of Irisin on the metabolic pathways of Treg cells may further affect the function and stability of these cells, such as enhancing the immune suppressive function by promoting fatty acid oxidation, which may subsequently affect neuroinflammation and cognitive function [89]. In different disease states, the role of Irisin may show differences, such as primarily exerting its function through immunomodulatory functions in autoimmune diseases, while in neurodegenerative diseases, it may exhibit more direct neuroprotective effects [90].

The choice of research methods and models is crucial for understanding the mechanism of action of Irisin. Different experimental settings may reveal different aspects of Irisin’s function. To resolve existing controversies and gain a deeper understanding of the molecular mechanisms of the interaction between Irisin and Treg cells, future research will need to adopt interdisciplinary approaches, combining knowledge from molecular biology, immunology, neuroscience, and metabolism. Clinical studies will further verify these mechanisms in humans and help develop new therapeutic strategies. With ongoing research, we will gain a more comprehensive understanding of the complex role of Irisin in immune regulation and neurocognitive function, providing new perspectives and strategies for the treatment of related diseases.

### 4.2. Controversies in the mechanism of action of Irisin and Treg in the regulation of neurocognitive function

Some studies propose that Irisin improves neurocognitive function by promoting the immunomodulatory action of Treg, while others suggest that Irisin directly affects the growth and function of neurons.

Research supporting the idea that Irisin improves neurocognitive function by enhancing Treg-mediated immunomodulation suggests that Irisin can increase the number and functionality of Treg, thereby regulating the immune system, reducing inflammatory responses and oxidative stress, and consequently improving cognitive function. These studies indicate that Irisin may promote the proliferation and differentiation of Treg and enhance their immunosuppressive function through pathways such as the activation of the JAK-STAT signalling pathway [91]. Additionally, Irisin may further strengthen the immunomodulatory role of Treg by modulating the secretion of cytokines, such as increasing the production of IL-10[128]. In the context of immune-mediated inflammation and neurodegenerative diseases such as multiple sclerosis (MS), Irisin holds potential for MS therapy by enhancing Treg functionality, thereby inhibiting the migration of effector T cells and autoreactive T cells into the central nervous system and consequently reducing myelin damage caused by these cells [7].

However, divergent research perspectives assert that Irisin exerts direct effects on neuronal growth and function, thereby playing a pivotal role in regulating neurocognitive functions. These studies highlight Irisin’s capability to stimulate neuronal growth and synaptogenesis, which enhances neuronal connectivity and subsequently improves cognitive processes like learning and memory. For example, recent scientific inquiries have elucidated Irisin’s ability to activate essential pathways such as the BDNF/TrkB signalling pathway, which is instrumental in promoting neuronal growth and fostering synaptogenesis [92]. Moreover, Irisin may also influence neurocognitive function by modulating neuronal excitability and synaptic plasticity [93]. In a mouse model of Parkinson’s disease, Irisin exerts neuroprotective effects by activating the Akt and ERK1/2 signalling pathways, thereby enhancing mitochondrial function [7]. Previous studies have shown that neurons and glial cells in the brains of male rats exhibit high levels of Irisin immunoreactivity [94]. In patients with multiple sclerosis (MS) and in the experimental autoimmune encephalomyelitis (EAE) mouse model, Irisin is localized within neurons, and its levels fluctuate with disease progression. These findings suggest that Irisin may directly participate in the pathological processes of MS/EAE, although the precise mechanisms remain to be elucidated [95].

Despite the inconsistencies in current research findings, it is reasonable to speculate that the interplay between Irisin and Tregs is complex and multifaceted, likely involving the coordinated action of multiple pathways and factors. For instance, Irisin enhances the immunomodulatory function of Tregs, thereby creating a favourable environment for neuronal health. Additionally, Irisin’s direct neurotrophic effects may further augment neurocognitive functions. Therefore, it is essential to conduct more comprehensive investigations to elucidate the precise mechanisms by which Irisin and Tregs modulate neurocognitive functions.

Future research could focus on several critical areas to advance our understanding of these interactions. Initially, exploring the intricate mechanisms of interaction between Irisin and Tregs, particularly how Irisin influences the immunomodulatory functions of Tregs and the specific pathways through which Tregs affect neurocognitive functions, would be crucial. Further, the direct effects of Irisin on neuronal growth and functioning warrant deeper investigation. This entails a comprehensive investigation into Irisin’s involvement in synaptogenesis and its impact on neuronal connectivity, pivotal for cognitive processing and maintaining brain health [96].

Understanding the interactions between Irisin and Tregs necessitates validation through rigorous experimentation, utilizing animal models and cellular assays. These investigative approaches are indispensable for elucidating the intricate regulatory effects exerted by Irisin on Treg functionality. Animal models allow for *in vivo* assessment, providing physiological context to observed outcomes. Additionally, cellular experiments offer mechanistic insights into the specific pathways and molecular mechanisms through which Irisin modulates Treg responses. Together, these methodologies contribute to a comprehensive understanding of Irisin’s role in immune regulation and its potential implications for neurocognitive functions. The application of animal models enables controlled experimentation to ascertain the physiological responses triggered by Irisin-Treg interactions. These studies not only validate the existence of these interactions but also delineate the physiological consequences within the immune system. Concurrently, cellular experiments offer a controlled environment to dissect the signalling pathways activated upon Irisin binding to Treg receptors. Such investigations are pivotal for characterizing the precise molecular events that underpin the regulatory effects of Irisin on Treg cells, providing foundational knowledge for therapeutic applications. The integration of findings from animal models and cellular experiments serves to substantiate the translational relevance of Irisin in neurocognitive functions. By examining how Irisin influences Treg-mediated immunomodulation across different experimental platforms, researchers can infer potential implications for cognitive processes. These insights are crucial for bridging the gap between basic research and clinical applications, offering prospects for therapeutic strategies targeting neuroinflammatory disorders associated with Treg dysfunction.

### 4.3. Future research directions and application prospects

Future studies should comprehensively assess the safety and long-term effects of Irisin therapy. While three-dimensional Alzheimer’s disease cell models demonstrate no toxicity at tested concentrations [83], clinical safety and efficacy data in both healthy populations and AD patients remain limited. Defining optimal dosing regimens represents a critical prerequisite for therapeutic translation. Irisin exerts positive effects on Treg function *via* multiple mechanisms, including modulating Treg cell differentiation and proliferation, enhancing their functionality, and increasing the production of anti-inflammatory cytokines [7]. We speculate that excessively high levels of Irisin may lead to overactivation of Treg cells, thereby weakening the body’s effective immune response to pathogens and increasing the risk of infection, or alternatively, disrupting immune homeostasis. Rigorous studies are needed to delineate the therapeutic window and mitigate such risks.

Additionally, Irisin’s metabolic effects have sparked concerns about possible metabolic dysregulation. Studies indicate Irisin improves insulin resistance in diabetic mice and regulates glucose [97] and lipid metabolism in human hepatocytes [98]. However, it is unknown whether Irisin treatment in neurocognitive impairment patients with normal blood glucose could trigger metabolic abnormalities. Blood-brain barrier penetration poses another challenge, limiting Irisin’s CNS bioavailability. Although nanoparticle delivery systems [99] may overcome this limitation, attempts to bypass the blood-brain barrier may introduce additional risks, such as unintended side effects or increased susceptibility to neuroinflammation. Thus, addressing these potential risks through rigorous preclinical and clinical studies is crucial to ensure the safety and efficacy of Irisin as a therapeutic agent.

The therapeutic potential of Irisin extends across multiple disease domains. In neurocognitive disorders, including AD and other dementias, Irisin may offer both symptomatic relief and disease-modifying effects through its neuroprotective properties [78]. Emerging evidence also suggests potential applications in autism spectrum disorders, depression, and anxiety, where modulation of neuroimmune pathways could provide novel treatment avenues [100]. In metabolic diseases such as obesity, metabolic syndrome, and diabetes, Irisin’s dual effects on energy metabolism and cognitive function position it as a unique therapeutic candidate.

To deepen our understanding of Irisin’s role in neurocognitive disorders and fully explore its therapeutic potential, future research should focus on mechanistic investigations to clarify how Irisin directly affects Tregs and the specific pathways through which it enhances neurocognitive function. Additionally, a critical and in-depth evaluation of the safety and long-term effects of Irisin-based therapies is essential. This includes extensive preclinical and clinical trials to determine an effective and safe dosing range, assess the risks associated with long-term treatment, and address potential side effects such as immune suppression and metabolic changes. Bridging the gap between preclinical findings and clinical application is a key step in translating Irisin treatment from bench to bedside. Establishing a clear correlation between Irisin levels, Treg function, and cognitive outcomes is crucial for understanding the therapeutic window of Irisin treatment and optimizing dosing strategies. The optimization of treatment protocols may be achieved through the development of targeted delivery systems, such as nanoparticle-encapsulated Irisin, to enhance the specificity and efficiency of Irisin delivery to target tissues [99], thereby reducing systemic exposure and associated risks. Although Irisin and Tregs offer new hope for the treatment of neurocognitive disorders, their clinical application depends on breakthroughs in mechanistic research, safety assessments, and innovations in delivery technologies.

## Summary

This review delves into the intricate interplay between Irisin and Tregs and their potential impact on neurocognitive functions. Irisin is a myokine released during physical activity and has been shown to significantly enhance both the quantity and functionality of Treg cells, indicating a key role in immune modulation. This interaction goes beyond simple immune cell regulation; Irisin influences Treg activity by modulating the differentiation and proliferation of these cells, providing a fresh perspective on its involvement in neurocognitive processes. Moreover, Irisin’s regulation of Treg cell metabolism opens new avenues for understanding its contributions to brain health and cognition.

At the neural level, Irisin has demonstrated potential to mitigate inflammation and oxidative stress, suggesting promising strategies for enhancing cognitive function and preventing neurodegenerative diseases such as Alzheimer’s and Parkinson’s. The protein also promotes neuronal growth and synaptogenesis, which are vital for cognitive processes including learning and memory, underscoring its beneficial effects in neurocognitive health.

Despite these promising findings, the precise mechanisms through which Irisin and Treg cells influence neurocognition remain a topic of debate and ongoing research. Some researchers propose that Irisin indirectly influences cognitive functions by boosting the immunomodulatory capabilities of Treg cells, while others suggest a more direct role where Irisin acts on neurons themselves.

The review highlights the need for further studies to unravel the specific interactions and mechanisms at play. Understanding these could lead to novel therapeutic targets and strategies for treating a spectrum of neurocognitive disorders, potentially shifting the paradigm in neurodegenerative disease management and cognitive health preservation. In summary, the dynamic interaction between Irisin and Treg cells is pivotal in neurocognitive regulation, and deepening our understanding of this relationship could unlock new possibilities for medical advancements in cognitive therapies and interventions.

## Data Availability

This article is a review of literature and does not contain any new data. Therefore, no data are available.

## Prior presentations

No presentation of work at conference meetings.
